# Identifying the predictors of time to diagnosis and treatment for people with cancer: a protocol for a cross-sectional survey supported by linked cancer registry data

**DOI:** 10.1136/bmjopen-2026-123201

**Published:** 2026-10-28

**Authors:** Hannah Jongebloed, Sharina Riva, Maria Bechelli, Sue Evans, Rebecca J Bergin, Anna Chapman, Tanita Botha, Helena Rodi, Sophie Boffa, Charlene Wright, Michele Conlin, Emily Lavcanski, Luc te Marvelde, Laura Alston, Anna Wong Shee, Patricia M Livingston, Victoria M White, Lan Gao, Skye Marshall, Anna Ugalde

**Affiliations:** 1 Institute for Health Transformation, Quality and Patient Safety, School of Nursing and Midwifery, Faculty of Health, Deakin University, Geelong, Victoria, Australia; 2 Victorian Cancer Registry, Cancer Council Victoria, Melbourne, Victoria, Australia; 3 Biostatistics Unit, Faculty of Health, Deakin University, Geelong 3220, Victoria, Australia; 4 Equitable Cancer Outcomes across Rural and Remote Australia (ECORRA) Consumer Advisory Committee, Deakin University, Geelong, Victoria, Australia; 5 Research Unit, Colac Area Health, Colac, Victoria, Australia; 6 Deakin Rural Health, Deakin University, Geelong, Victoria, Australia; 7 Grampians Health, Ballarat, Victoria, Australia; 8 Faculty of Health, Deakin University, Geelong, Victoria, Australia; 9 School of Psychology, Faculty of Health, Deakin University, Geelong, Victoria, Australia; 10 Institute for Health Transformation, Deakin Health Economics, Deakin University, Geelong, Victoria, Australia

**Keywords:** ONCOLOGY, Health Services, Health Equity

## Abstract

**Abstract:**

**Introduction:**

People from rural and remote communities experience poorer cancer outcomes compared with their urban counterparts. In Australia, the Optimal Care Pathways provide guidance for best practice cancer care and outline optimal processes and time frames for diagnosis and treatment. Evidence indicates that survival is improved when care is aligned to these time frames. Understanding the predictors of delays in meeting these benchmarks is therefore critical to improving equity in cancer outcomes. However, the predictors of such delays, particularly in rural and remote areas, remain poorly understood.

This paper describes the protocol of a study that aims to identify predictors of diagnostic and treatment time frame alignment and unwarranted delays according to the Optimal Care Pathways for people with cancer in rural compared with urban areas.

**Methods and analysis:**

This cross-sectional study uses a statewide survey supported by linked Victorian Cancer Registry data. Participants are identified via the Victoria Cancer Registry and are eligible if ≥18 years and diagnosed with head or neck, colorectal, lung, melanoma, prostate or oesophageal cancer in the past 12 months. The survey is disseminated via mail and collects self-report data on cancer diagnostics, treatments and experiences. Respondents’ data are linked with Victorian Cancer Registry data elements, including hospital, pathology and demographic information. One thousand completed surveys are anticipated (500 rural; 500 urban). The primary outcomes are time to diagnosis and time to treatment commencement. Regression models will be used to assess the effect of rurality status and selected predictors. Interaction terms between rurality status and key predictors will also be included. For each outcome, models will initially incorporate all six cancer types. This project will move beyond describing inequities to identifying the key factors associated with time to diagnosis and treatment, informing the generation of targeted solutions to optimise cancer outcomes, regardless of where people live.

**Ethics and dissemination:**

This study was reviewed and approved by the Cancer Council Victoria Human Research Ethics Committee (ref: HREC #2416). Findings will be disseminated through peer-reviewed publications and scientific conference presentations.

**STRENGTHS AND LIMITATIONS OF THIS STUDY**

- A strength is the recruitment approach through a population-based cancer registry, which ensures random sampling from population-wide data, and all participants have a confirmed diagnosis of cancer.
- A strength of this study is the linking of cancer registry data with self-report experiences to identify both predictors of delays to and alignment with diagnosis and treatment time frames, as defined by the Optimal Care Pathways.
- A further strength is the alignment with a national and international focus on equitable cancer care. Subgroups in need of specific policy and research attention will be identified; as will points for intervention across the diagnostic and treatment pathways.
- Limitations include the reliance on patient self-report, a focus on the most recent cancer type when some people may have multiple diagnoses, the exclusion of rare and less common cancer types and people who died within the first 12 months post diagnosis, and the recruitment through a single state.

## Background

 Cancer is a major health challenge, with the number of newly diagnosed cases worldwide projected to reach 32.6 million annually by 2045.^1^ People from rural and remote communities experience poorer cancer outcomes, with 10% lower odds of cancer-specific survival and 15% lower odds of all-cause survival compared with urban populations.^2^ Although health and healthcare disparities affecting rural and remote areas are widely recognised,^3^ the drivers and underlying reasons for these inequities are less understood. The factors contributing to poorer survival outcomes are likely to be multifaceted and complex.^4^

People living in rural and remote areas experience delays to diagnosis and treatment commencement.^3 5 6^ There are several complexities in their care across the cancer trajectory. People from rural and remote areas can experience reduced access to and engagement with cancer screening programmes compared with those from metropolitan areas.^7 8^ Travel can be prohibitive in accessing best practice care, due to distance and cost. Time away from essential duties and the requirements of employment (farming, agriculture) may be a further barrier.^4^ Radiotherapy is a common treatment for several types of cancer; it is commonly delivered daily over several weeks, in regional or metropolitan centres, and uptake is lower for people from rural areas. Travel costs not only the patient’s time and cost of travel, but also the time and costs of carers/family that may accompany them, costs associated with accommodation that may be needed and potentially forgone wages.^9 10^ In many rural and remote areas, access to allied health practitioners and oncologists is reduced.^11–13^ Given these challenges, understanding how to deliver best practice cancer care in population groups that are known to have poorer outcomes is a priority.^14 15^

Robust and accurate data are essential components of understanding cancer inequities. In Australia, the Optimal Care Pathways (OCPs) are frameworks for best practice cancer care^16^ and are nationally endorsed as part of the Australian Cancer Plan.^17^ OCPs have been developed for most cancer types and several population groups, outlining expectations for quality, safe cancer care including diagnostic and treatment time frames.^18^ An observational study using linked, routinely collected data which included over 15 000 people with colon cancer found improved outcomes when cancer care was aligned with the OCPs.^19^ Similarly, risk of death for women with breast cancer in Victoria, Australia was found to be lower for women whose treatment aligned with the OCP for people with breast cancer.^20^ A meta-analysis showed treatment delay across the care pathway as a major issue, with delays of 4 weeks associated with increased mortality for a range of cancers.^21^

Despite this, there is some evidence that the recommended time frames for diagnosis and treatment are often not met. For example, in several studies including Australian samples, key benchmarks were observed as being difficult to achieve, particularly in the interval between general practitioner referral and diagnosis.^22–24^ Assessing the diagnostic and treatment intervals for people from rural areas with cancer can identify where variations in alignment with care pathways occur. This evidence is needed to determine the most appropriate interventions to address the reason for the variation, for instance rapid access diagnostic clinics, hub-and-spoke models, telehealth and patient navigation.

There is a need to consider how to implement interventions to deliver best practice cancer care, but without an understanding of factors associated with delays to diagnosis and treatment, there is a lack of evidence to underpin policy initiatives or local programmes. This paper describes the protocol of a study that aims to identify predictors of diagnostic and treatment time frame alignment and unwarranted delays, according to the OCP, for people with cancer in rural compared with urban areas of Victoria, Australia.

## Methods

### Study design

This cross-sectional study uses a state-wide survey supported by a linked state-based registry dataset. Data collection commenced in March 2025 and will continue until the end of May 2026. This study has been reported according to the Strengthening the Reporting of Observational studies in Epidemiology (STROBE) checklist for cross-sectional studies.^25^

### Study setting

The setting for this study is Victoria, a state of Australia with a population of over 7 million people,^26^ representing approximately 26% of the country’s population.^27^ The state spans approximately 227 444 km^2^,^28^ and about 25% of the Victorian population lives outside metropolitan areas.^29^

### Participants

Participants are eligible if they are aged 18 years or over, reside in Victoria, Australia, and have a diagnosis date between 1 January 2024 and 28 February 2025. All cases approached are reviewed by Victorian Cancer Registry (VCR) staff and are required to have been diagnosed with an eligible cancer type within the previous 12 months, to minimise recall bias. Eligible cancer sites were classified using International Classification of Diseases, 10th Revision^30^ codes, including colorectal cancer (C18–C20, excluding C18.1), oesophageal cancer (C15), head and neck cancers (C00–C14), lung cancer (C34), cutaneous melanoma (C43, excluding in situ lesions) and prostate cancer (C61). These tumour types were selected due to the evidence of survival discrepancies between metropolitan and rural Victorian patients with cancer.^31^

Individuals are excluded if they have insufficient or no pathology on record to confirm cancer diagnosis, if their current address is overseas, interstate or in an institution such as an aged care facility. Prior to mailing the survey and reminders, a vital status check is performed by the VCR at the beginning of each month against the Victorian Death Registry; those who have died are excluded from being eligible for approach. The VCR is a department within Cancer Council Victoria, that collects information on all cancers diagnosed in Victorian residents on behalf of the Victorian Department of Health, aligning with legislative requirements (Improving Outcomes Act 2014).^32^

### Recruitment

Recruitment is coordinated by the VCR. This study adopts active opt-in consent, with a consent form completed concurrently with the voluntary completion and return of the survey. Following participant consent, the VCR releases a defined data extract for that individual to the research team and links survey responses to registry variables using the registry’s unique identifier (deterministic linkage).

Eligible participants are identified via rolling recruitment over the 14-month period through the VCR. A study pack is distributed by postal mail which includes a cover letter, patient information sheet, consent form, study-specific questionnaire, reply-paid envelope, information brochures on Cancer Information and Support services, and the VCR. A QR code is provided to enable online survey completion. Non-respondents are mailed a single reminder 3 weeks after the original survey distribution, consisting of a postcard with the QR code and a prompt to complete the survey.

### Data collection and outcomes

Data collection comprises two components: Survey data and linked registry data. A study-specific questionnaire was developed through adaptation of selected items from existing validated questionnaires including the International Cancer Benchmarking Partnership (ICBP) questionnaire, the National Health Service England National Cancer Patient Experience Survey and the Victorian Healthcare Experience Survey^33–36^ with iterative refinement through consumer and investigator input. The questionnaire has 58 items and collects self-reported data on cancer diagnosis and treatments (including events leading to diagnosis, dates of first symptoms and healthcare appointments to discuss symptoms if applicable, number of engagements with primary healthcare, engagement with cancer screening programmes, self-reported dates of diagnosis and treatment commencement), experiences (including financial barriers and travel requirements associated with treatment commencement) and sociodemographics (online supplemental file 1). Data elements released from the VCR for those who consent include hospital (date of admission), pathology (cancer type, primary site (International Classification of Diseases for Oncology, 3rd Edition (ICDO-3)), morphology, tumour behaviour and registry-derived stage) and demographic information (sex, age at diagnosis, date of birth, year of death (if applicable), country of birth, aboriginality, current address, address at diagnosis, index of socio-economic disadvantage (Socio-Economic Indexes for Areas), rurality (Australian Statistical Geography Standard and Modified Monash Model (MMM)).

The primary outcomes are time (assessed in days) to (1) time from initial presentation to cancer diagnosis and (2) time from definitive diagnosis to start of first treatment. Secondary outcomes are whether time frames for cancer care aligned with OCP time frames specified for each cancer type.

The primary exposure is rurality status as per the Rural–Urban Classification System Harmonisation Framework, with the Modified Monash Model selected as the rurality classification system most appropriate.^37^ The Rural–Urban Classification System Harmonisation Framework provides a consistent five-point rurality scale and standardised urban–rural dichotomisation to standardise categorisation and reporting of rurality. For full details of the classification system, please refer to the original publication.^37^ Rurality is dichotomised using the Modified Monash Model with residential postcodes classified as MMM1-2 (metropolitan and regional centres) being ‘urban’ and MMM3-7 (large rural towns, medium rural towns, small rural towns, remote communities and very remote communities) being ‘rural’.^37^ Potential predictors of interest include age, sex, smoking status, cancer type, country of birth, languages other than English spoken at home, registry-derived stage, number of appointments prior to diagnosis, treatment type, socio-economic status, highest level of education, employment status and private health insurance status.

### Sampling

A formal, a priori sample size calculation was not performed. Instead, the sample size was determined pragmatically based on available funding and recruitment feasibility, taking into account the recruitment time frame required and the number of cases diagnosed in Victoria for each cancer type. As this was an observational study, rather than a clinical trial, a pragmatic sample size approach was considered appropriate. We targeted balanced group sizes to facilitate comparisons with an expected 500 participants in the rural group and 500 participants in the urban group and equal numbers across cancer types, except for oesophageal cancer due to its lower incidence rate. Within each rurality stratum and cancer type, simple random sampling without replacement is applied to the monthly eligible list to achieve target accruals. Based on previous studies where recruitment was undertaken by the VCR, it is estimated that consent rate will vary by cancer type, with an average anticipated consent rate of 20–30%.^3 24^

### Data analysis

Time intervals of the primary outcomes will be calculated in accordance with the Aarhus statement for cancer diagnosis research.^38^ The Aarhus statement provides a standardised framework for designing, conducting and reporting research on cancer diagnosis, definitions and measurement recommendations for key time points and intervals to cancer diagnosis and treatment. For full details, please refer to the original publication.^38^ Where there are missing or incomplete data, or multiple responses, we will apply the ICBP Module 4 data coding rules, which provide a hierarchy for information sources.^23^ For full details, please refer to the original publication.^23^ The diagnostic interval will be calculated as the number of days between the reported date of a patients first appointment with a health professional about their health concern/s or symptom/s (self-reported) and the date of diagnosis (supplied by the VCR). The treatment interval will be calculated as the number of days between the date of cancer diagnosis and the date the patient received their first treatment of any intent (self-reported).

The statistical analysis plan includes a comprehensive descriptive overview of all data collected, along with appropriate data quality checks. Descriptive statistics will be tailored to the underlying distribution: continuous variables will be summarised using means and SD or medians and IQRs for skewed data; and categorical variables using counts and percentages. All demographic characteristics and potential predictors of interest will be summarised and stratified by rurality status groups, and the differences between these groups will be explored. This will include tests such as the χ² (or Fisher’s exact test) for categorical variables and the independent t-test (or the non-parametric alternative) for continuous data.

For the primary outcomes of interest (time to diagnosis and time to treatment commencement expressed in days) regression models will be used to assess the effect of rurality status and selected predictors. Interaction terms between rurality status and key predictors will be included. For each outcome, models will initially incorporate all cancer types. Additional logistic regression models will examine whether a delay occurred (yes/no), defined as alignment with the recommended OCP time frame for each cancer type. Proportions of participants receiving care within OCP time frames will be reported by cancer type.

Statistical significance will consider measures of precision, the effect size and p values. Analyses will be conducted using R V.4.3.2^39^ and Microsoft Excel.

### Ethics, governance and dissemination

This study has been approved by the Cancer Council Victoria Human Research Ethics Committee (ref: HREC #2416). Consent includes access to the participants’ VCR data. Participants were not reimbursed for participation in this study.

Study governance comprises a steering committee and project working group. The steering committee includes all investigators and provides input at key project points (eg, survey development, interpretation of results) as needed via meetings or email. The project working group (HJ, SR, MB, SE and AU) conduct, manage and monitor recruitment, mail-outs, ethics, data handling and budget. This study is part of the Equitable Cancer Outcomes across Rural and Remote Australia (ECORRA) Research Programme, a national, transdisciplinary effort to understand and reduce inequities in cancer outcomes across rural and remote areas of Australia.^40^ The programme is underpinned by a fit-for purpose governance structure to ensure transparency and coordination, a genuine rural and remote focus, embedded consumer involvement, cross-sector partnerships beyond academia and place-based engagement that tailors initiatives to local contexts.

Findings will be disseminated through peer-reviewed publications and scientific conference presentations.

### Patient and public involvement

Embedded consumer involvement is a principle of this research programme, and a diverse consumer panel was launched to guide this and other ECORRA projects.^40^ Consumers are defined as people with lived experience of cancer as a patient or carer. All panel members have lived experience of rural cancer care, whether through making decisions about treatment location, receiving treatment in rural communities or travelling to metropolitan centres. Consumers contributed to the development of the research concept and grant proposal, reviewed the questionnaire, informed the methodology and are active members on the steering committee. They will be integral to the interpretation of results. A consumer is a coauthor of this protocol.

## Discussion

Understanding unwarranted variations in cancer care can inform targeted initiatives and interventions; and representative data are an essential component of understanding cancer inequities.^15^ This study has several strengths. Registry-based recruitment ensures all participants have a confirmed diagnosis of cancer, unlike social media recruitment where eligibility cannot be verified. Recruiting participants through the population-based cancer registry enabled random sampling from population-wide data as the VCR captures all Victorians diagnosed with cancer. Registry infrastructure ensures that potential participants are not excluded due to delays in diagnostic information, and data linkage minimises the risk of deceased people being approached. Capturing a broad cross-section of patients with cancer ensures that findings are not limited to patients attending a specific treatment centre or residing in one regional area.

A further strength is the study’s integration of self-reported cancer experiences, with linked clinical variables including date of diagnosis, demographics and staging. The inclusion of multiple cancer types allows for analysis to identify whether predictors of delays differ by geographical region and by cancer type. Comparisons will be able to be conducted between the clinical variables recorded by the registry and by the recall of individual people affected by cancer. Participants also have the option of leaving their details for future research, pending ethics approval, allowing for a sample to be approached for future work.

The mail-out and reminder processes have enabled a more inclusive and reliable sample; however, this is resource intensive. The process to achieve this includes the mailout of 300–400 recruitment packs and 200–300 reminders per month, which are manually compiled and posted. We decided to post reminders, rather than post the full survey a second time, to reduce postage costs and paper waste. The collaboration with the cancer registry is essential to both the mailout and the recruitment strategy, and in ensuring expert advice, appropriate methods and criteria are used across the project. Four collaborators and coauthors either work within or are experienced in the registry. The VCR prepares a monthly report which documents recruitment progress across cancer types and rural and urban categories, against timelines.

The limitations of this study include the reliance on patient self-report with a survey. While there will be opportunity to compare patient self-report with the data in the registry, several data points, including date of first symptom/s and date of first appointment with a health professional are entirely contingent on patient self-report. Additionally, participants are provided with instructions that the questions relate to their most recent cancer diagnosis; however, people with multiple cancer diagnoses may find answers around date of diagnosis and dates of treatments difficult to answer. Recruiting participants 12 months after diagnosis may have introduced bias, as individuals who died during this period were ineligible and some approached may have been too unwell to participate. Additionally, disclosure of the study aim may have influenced responses to the self-report questionnaire.

The exclusion of rare cancer types is another limitation and informed by resourcing, both in the funding available and in the time frames for recruitment. Rare and less common cancers would take longer to recruit required sample sizes for inclusion in the model. We attempted to include people with ovarian cancer in this study; however, recruitment rates were too low for inclusion in the model. Data collection of women with ovarian cancer is continuing and will be reported separately. A further limitation is that this study is also being conducted entirely in one state. Other states in Australia, including Northern Territory, Western Australia, South Australia and Queensland, have more remote and very remote regions. Expansion to other states and territories, and more remote communities, is an avenue for future research. Finally, it is possible that all factors that may be associated with the outcomes of interest are not captured within this study.

This study will provide a detailed understanding of the factors associated with delays to diagnosis and delays to treatment and identify subgroups receiving care less aligned with OCPs. Findings may inform subsequent work with clinicians, consumers, health service leaders and policymakers to develop policy or research initiatives addressing gaps in care. Rapid evidence reviews and health economic analysis could appraise potential interventions. The publication of the survey instrument (online supplemental file 1) supports scalability to other cancer types, regions or countries. Overall, this work represents a unique and important contribution to understanding and addressing the complex and challenging issues contributing to poorer cancer outcomes for rural and remote communities.^40^

This study links cancer registry data with self-report experiences to identify predictors of delays to diagnosis and treatment, and alignment with best-practice cancer care frameworks. This project delivers on a national and international focus on equitable cancer care and will identify subgroups in need of specific policy and research attention and points for intervention across the diagnostic and treatment pathways. It will add to the body of literature that helps to inform the generation of evidence-based solutions to drive better cancer outcomes, regardless of where people live.

## Supplementary material

10.1136/bmjopen-2026-123201 online supplemental file 1
