# Supplementary material for: Identifying the predictors of time to diagnosis and treatment for people with cancer: a protocol for a cross-sectional survey supported by linked cancer registry data
Source: BMJ Open. 2026 Oct 28;16(10):e123201. doi: 10.1136/bmjopen-2026-123201 (PMC13723840; doi:10.1136/bmjopen-2026-123201)
Supplement: Supplementary file 1 [file bmjopen-2026-123201.reviewer_comments.pdf]

PEER REVIEW HISTORY

BMJ Open publishes all reviews undertaken for accepted manuscripts. Reviewers are asked to complete a checklist review form and are provided with free text boxes to elaborate on their assessment. These free text comments are reproduced below.

ARTICLE DETAILS

Title (Provisional)

Identifying the predictors of time to diagnosis and treatment for people with cancer: A protocol for a cross-sectional survey supported by linked cancer registry data

Authors

Jongebloed, Hannah; Riva, Sharina; Bechelli, Maria; Evans, Sue; Bergin, Rebecca J; Chapman, Anna; Botha, Tanita; Rodi, Helena; Boffa, Sophie; Wright, Charlene; Conlin, Michele; Lavcanski, Emily; te Marvelde, Luc; Alston, Laura; Wong Shee, Anna; Livingston, Patricia M; White, Victoria M.; Gao, Lan; Marshall, Skye; Ugalde, Anna

---

VERSION 1 - REVIEW

**Reviewer**	**1**
**Name**	**Woodward, Emma R.**
**Affiliation**	**Manchester University NHS Foundation Trust**
**Date**	**27-Jul-2026**
**COI**	**none**

This manuscript is, for the most part, well written, setting out the rationale for the study. I have some comments for suggested amendments:

P6, Line 42 - sentence beginning, ‘They outline….’ – would benefit from re-writing to ensure clarity

P8, Line 56 – need to make more please of these confounders – they are an inevitable limitation of questionnaire based studies

P11, Line 20 - sentence beginning, ‘A further strength…..’ – would benefit from re-writing to ensure clarity

In terms of methods, I think this manuscript warrants a paragraph on clearly describing what data has to be self-reported (through the questionnaire) versus what data is registry collected. Readers not being familiar with the healthcare system within which the authors operate would benefit from this detail, as it helps clarify where a questionnaire approach is needed, and where the more objective medical record data/registry data can be used.

**Reviewer**	**2**
**Name**	**Kiemeney, Lambertus**
**Affiliation**	**Radboud Universiteit, Dept for Health Evidence**
**Date**	**17-Aug-2026**
**COI**	**none**

I have reviewed the paper aiming at assessing clarity of the protocol, not validity of the protocol, in line with the publication type. I have several comments about the validity but I ignored these for my advice about acceptance of the paper.

Comments:

1. Why is it necessary to have 20 authors on a protocol paper? I would suggest to only include the (really) 'responsible' authors.

2. The authors claim they will come up with 'robust and accurate' results. I doubt that. With only 1000 participants, 6 different tumor sites and quite a few potential determinants for time to diagnosis and time to treatment, a claim of robust and accurate results cannot be made in my opinion. There is no formal power analysis but a small simulation study of the standard errors around estimators would be needed for such a claim. If not, remove this claimed strength throughout the paper.

3. The authors state "This project will move beyond describing inequities to identifying the key drivers …". The paper is mixing prediction research (see e.g. title) and causality research (e.g. the use of the words 'drivers' and 'confounders') objectives constantly. Because their ultimate aim is to improve clinical care by taking away causes for delay, obviously their intent is causation This should be made clearer.

4. It is stated that "A strength is the recruitment approach through a population-based cancer registry". I agree but there are also negative consequences of this design. First: because participants are recruited who are diagnosed less than one year ago, some patients (especially patients with oesophageal or lung cancer) may have died already (or may be too ill) before recruitment, i.e. potential survival bias. Also, the fact that patients know the aims of the study, may have influenced the information they provide (information bias). Another design might have been difficult but at least mention these potential weaknesses.

5. Page 6 line 25: what makes the survey "state-wide" with only 1000 participants?

6. According to protocol, data collection has ended already (end of May 2026). The policy of the journal is not to accept such papers.

7. "Individuals are excluded …. if their current address is …, interstate, or in an institution such as an aged care facility." Explain why.

8. Page 7 line 4: Be more clear what "those cases" are (deaths).

9. The postal mail was, apparently, very labor-intensive. Why was not chosen for an online questionnaire (maybe after a brief postal invitation) with an option to request a paper questionnaire instead of the other way around?

10. "… selected items from existing validated questionnaires". There is a reference to these questionnaires but it might be good to mention them in the main text.

11. Explain better "Secondary outcomes are alignment with OCP timeframes for each tumour stream."

12. "The primary exposure is rurality status as per the Rural-Urban Classification System Harmonisation Framework, with the Monash Modified Model (MMM) selected as the rurality classification system most appropriate.37". Explain better what this model is. How are the different categories defined (e.g. when is a large rural town large?).

13. Instead of working only with urban versus rural why not do the authors analyze physical distance to the nearest hospital that can diagnose (and treat) cancer. This would provide some idea whether it is distance or other factors (such as rural-type professions) that cause the delays.

14. It is obvious why some characteristics are considered to be potential effect modifiers. This is less clear to me for stage and treatment type. Please explain.

15. "Potential confounders include socioeconomic status, highest level of education, employment status and private health insurance status." What about e.g., relationship situation. This is a protocol paper. So please be complete. I suggest to include a graphical display in which you show which variables are considered independent variables, dependent variables, EM, and confounders. You may also include a DAG but I don't think that is better.

16. Please explain short what the Aarhus statement is beyond "a standardized framework for designing, conducting, and reporting research on cancer diagnosis, aimed at improving the measurement of diagnostic pathways and time intervals."

17. The same for the ICBP Module 4 data coding rules.

18. "Interaction terms between rurality status and key predictors will be included." Because researchers are inclined to remove interaction terms that are not statistically significant, you don't get a good idea of different situations in different main groups. Please consider stratified analysis in rural and urban groups as an alternative.

19. Poisson regression. It is not entirely clear to me why you use this instead of logistic regression.

20. Page 10 line 20: remove "of".

21. Page 10 line 24: what are "delayed predictors"?

VERSION 1 - AUTHOR RESPONSE

Reviewer #1

Comments to the Author:
This manuscript is, for the most part, well written, setting out the rationale for the study. I have some comments for suggested amendments:

Comment 1: P6, Line 42 - sentence beginning, 'They outline….' – would benefit from re-writing to ensure clarity

> ***Response:*** *Thank you for the reflections on the writing quality of this manuscript and we agree this sentence could be better presented. We have rewritten to: "OCPs have been developed for most cancer types, and several population groups, outlining expectations for quality, safe cancer care including diagnostic and treatment timeframes.[18]" (page 4, lines - 117-119).*

Comment 2: P8, Line 56 – need to make more please of these confounders – they are an inevitable limitation of questionnaire-based studies

> ***Response:*** *As per the feedback from both reviewers, these variables have now been reframed as potential predictors of interest. Please see below for the response to reviewer 2, comment 15. We have also included the possibility of unmeasured factors to influence the observed associations as a limitation: "Finally, it is possible that all factors that may be associated with the outcomes of interest are not captured within this study" (pg 11, lines 362-364)*

Comment 3: P11, Line 20 - sentence beginning, 'A further strength…..' – would benefit from re-writing to ensure clarity

> ***Response:*** *This has also been rewritten: "The inclusion of different multiple cancer types, which allows for analysis to identify whether predictors of delays differ by both geographic region and cancer type." (page 10, lines 325-327)*

Comment 4: In terms of methods, I think this manuscript warrants a paragraph on clearly describing what data has to be self-reported (through the questionnaire) versus what data is registry collected. Readers not being familiar with the healthcare system within which the authors operate would benefit from this detail, as it helps clarify where a questionnaire approach is needed, and where the more objective medical record data/registry data can be used.

> ***Response:*** *Thank you for this comment and this inclusion has strengthened the work. We now clearly state that the survey (and Supplementary File 1) is all self-reported, and provide further details of the data provided by the registry: "(including events leading to diagnosis, dates of first symptoms and healthcare appointments to discuss symptoms if applicable, number of engagements with primary health care, engagement with cancer screening programs, self-reported dates of diagnosis and treatment commencement), experiences (including financial barriers and travel requirements associated with treatment commencement), and socio-demographics (Supplementary File 1). Data elements released from the VCR for those who consent include hospital (date of admission), pathology (tumour type, primary site (ICDO-3), morphology, tumour behaviour and registry-derived stage) and*

demographic information (sex, age at diagnosis, date of birth, year of death, country of birth, aboriginality, current address, address at diagnosis, index of socio-economic disadvantage (Socio-Economic Indexes for Areas (SEIFA)), rurality (Australian Statistical Geography Standard (ASGS) and Modified Monash Model (MMM)).” (pg 6 & 7, lines 196 – 208)

Reviewer #2

Comments to the Author:

I have reviewed the paper aiming at assessing clarity of the protocol, not validity of the protocol, in line with the publication type. I have several comments about the validity but I ignored these for my advice about acceptance of the paper.

Comments:

Comment 1: Why is it necessary to have 20 authors on a protocol paper? I would suggest to only include the (really) ‘responsible’ authors.

> ***Response:** We respectfully note that all authors fulfill authorship requirements, and the authorship reflects a transdisciplinary team, representing academics from a range of disciplines, clinicians and health service managers, consumers, statisticians, and early career researchers who have been integral to the development of the project and protocol. Given the inputs of all authors into this manuscript, removing authors is not appropriate.*

Comment 2: The authors claim they will come up with ‘robust and accurate’ results. I doubt that. With only 1000 participants, 6 different tumor sites and quite a few potential determinants for time to diagnosis and time to treatment, a claim of robust and accurate results cannot be made in my opinion. There is no formal power analysis but a small simulation study of the standard errors around estimators would be needed for such a claim. If not, remove this claimed strength throughout the paper.

> ***Response:** It is important to contextualise the sample size: this is a survey which describes the cancer experience, linked to cancer registry data. It is not positioned as an epidemiological study, where sample sizes are larger, but the detail obtained will be more in depth. We have removed this as a strength.*

Comment 3: The authors state “This project will move beyond describing inequities to identifying the key drivers …”. The paper is mixing prediction research (see e.g. title) and causality research (e.g. the use of the words ‘drivers’ and ‘confounders’) objectives constantly. Because their ultimate aim is to improve clinical care by taking away causes for delay, obviously their intent is causation This should be made clearer.

> ***Response:** As this is a cross-sectional study, the aim is to identify variables associated with time to diagnosis and treatment, rather than identify casual determinants. We have revised our language throughout to more accurately reflect this.*

Comment 4: It is stated that “A strength is the recruitment approach through a population-based cancer registry”. I agree but there are also negative consequences of this design. First: because participants are recruited who are diagnosed less than one year ago, some patients (especially patients with oesophageal or lung cancer) may have died already (or may be too ill) before

recruitment, i.e. potential survival bias. Also, the fact that patients know the aims of the study, may have influenced the information they provide (information bias). Another design might have been difficult but at least mention these potential weaknesses.

> ***Response:*** *These have been included as weaknesses as follows: "Recruiting participants 12 months after diagnosis may have introduced bias, as individuals who died during this period were ineligible and some approached may have been too unwell to participate. Additionally, disclosure of the study aim may have influenced responses to the self-report questionnaire." (pg 10, lines 348-352)*

Comment 5: Page 6 line 25: what makes the survey "state-wide" with only 1000 participants?

> ***Response:*** *This is statewide as it is conducted across the state of Victoria in Australia and all people with a cancer diagnosis across the state are considered for participation. Recruitment is not restricted to a particular hospital or network of hospitals.*

Comment 6: According to protocol, data collection has ended already (end of May 2026). The policy of the journal is not to accept such papers.

> ***Response:*** *We will allow the editor to make this decision, respectfully noting we fulfilled all requirements of the journal at the time of submission.*

Comment 7: "Individuals are excluded …. if their current address is …, interstate, or in an institution such as an aged care facility." Explain why.

> ***Response:*** *The registry has data for cases within the state, and therefore only approaches Victorian residents. For people living outside of Victoria, it can be more challenging to maintain accurate contact details, undertake appropriate follow-up and apply recruitment procedures consistently. Aged care facilities deliver very different healthcare to people in the general population, and are excluded from approach to reduce the risk of approaching individuals who may have significant frailty, cognitive impairment or other circumstances that could affect their ability to provide informed consent. In addition, recruitment approaches in these settings may inadvertently disclose sensitive health information to carers, family members, or facility staff, creating potential privacy concerns.*

Comment 8: Page 7 line 4: Be more clear what "those cases" are (deaths).

> ***Response:*** *We have edited this sentence to be clearer and it now reads: "Prior to mailing the survey and reminders, a vital status check is performed by the VCR at the beginning of each month against the Victorian Death Registry; those who have died are excluded from being eligible for approach." (pg 6, lines 170-171).*

Comment 9: The postal mail was, apparently, very labor-intensive. Why was not chosen for an online questionnaire (maybe after a brief postal invitation) with an option to request a paper questionnaire instead of the other way around?

> ***Response:*** *Thank you for this comment. The design drew upon the past experiences with registry studies, and we are confident this suggestion would have led to decreased consent rates. Interestingly, despite having the option to complete the study online, 85% of*

participants returned the paper survey instead of completing the online version. Without email addresses or phone numbers of participants, mail was the only way to make contact.

Comment 10: "… selected items from existing validated questionnaires". There is a reference to these questionnaires but it might be good to mention them in the main text.

> ***Response:*** *These are mentioned in the methods section, and have been expanded on, where we state: "Data collection comprises two components: Survey data and linked registry data. A study-specific questionnaire was developed through adaptation of selected items from existing validated questionnaires including the International Cancer Benchmarking Partnership questionnaire, the National Health Service England National Cancer Patient Experience Survey and the Victorian Healthcare Experience Survey[33-36] with iterative refinement through consumer and investigator input." (pg 7, lines 192-196).*

Comment 11: Explain better "Secondary outcomes are alignment with OCP timeframes for each tumour stream."

> ***Response:*** *This has been edited: "Secondary outcomes are whether timeframes for cancer care aligned with OCP timeframes specified for each tumour type."*

Comment 12: "The primary exposure is rurality status as per the Rural-Urban Classification System Harmonisation Framework, with the Monash Modified Model (MMM) selected as the rurality classification system most appropriate.37". Explain better what this model is. How are the different categories defined (e.g. when is a large rural town large?).

> ***Response:*** *Thank you for this response, this is fully detailed in the paper which is referenced, and as this paper is quite long for a cross sectional protocol at >3000 words, we have briefly included a description, stating: "The Rural-Urban Classification System Harmonisation (RUSCH) Framework provides a consistent five-point rurality scale and standardised urban-rural dichotomisation, to standardise categorisation and reporting of rurality". We have also added: "For full details of the classification system, please refer to the original publication.[37]" (pg. 7, lines 210-214).*

Comment 13: Instead of working only with urban versus rural why not do the authors analyze physical distance to the nearest hospital that can diagnose (and treat) cancer. This would provide some idea whether it is distance or other factors (such as rural-type professions) that cause the delays.

> ***Response:*** *Where an individual undertakes their cancer care is influenced by a range of factors including service location, visiting medical specialist availability, cancer type and stage, and patient preference. Classifications of rurality as determined by eligible geographical classification systems within the RUSCH Framework consider these complex factors, including physical distance, collectively. We acknowledge that the data collected through this project includes patient address at diagnosis, and for a subset of the sample (those who have undergone treatment), treatment location (suburb) and this may allow for further analysis of the relationship between distance and outcomes.*

Comment 14: It is obvious why some characteristics are considered to be potential effect modifiers. This is less clear to me for stage and treatment type. Please explain.

Response: *Patients with early-stage cancers may enter the diagnostic pathway through screening or investigation of relatively subtle symptoms. Rural patients may face greater geographic barriers to screening, diagnostic imaging, specialist assessment, or referral, potentially producing a larger rural–urban difference in time to diagnosis. However, for advanced stage disease, symptoms may be more pronounced and generate more urgent investigation, potentially reducing the rurality-related difference in diagnostic delay. Alternatively, advanced disease may require more complex specialist investigations and multidisciplinary care, meaning that rural patients could experience greater delays because of limited access to specialised services. We have reframed these as potential predictors for inclusion in the regression model (see response to comment 15 below).*

Comment 15: "Potential confounders include socioeconomic status, highest level of education, employment status and private health insurance status." What about e.g., relationship situation. This is a protocol paper. So please be complete. I suggest to include a graphical display in which you show which variables are considered independent variables, dependent variables, EM, and confounders. You may also include a DAG but I don't think that is better.

Response: *Thank you for this comment. These variables have been reframed to align with the main aim, to identify predictors of time to diagnosis and treatment for people with cancer and now reads as follows: "Potential predictors of interest include age, sex, smoking status, cancer type, country of birth, languages other than English spoken at home, registry derived stage, number of appointments prior to diagnosis, treatment type, socioeconomic status, highest level of education, employment status and private health insurance status." (pg 7, lines 223-228)*

Comment 16: Please explain short what the Aarhus statement is beyond "a standardized framework for designing, conducting, and reporting research on cancer diagnosis, aimed at improving the measurement of diagnostic pathways and time intervals."

Response: *We have expanded upon our description, and referred readers to the full paper should they wish to review it further: "The Aarhus statement provides a standardized framework for designing, conducting, and reporting research on cancer diagnosis, including definitions and measurement recommendations for key time points and intervals to cancer diagnosis and treatment. For full details, please refer to the original publication.[38]" (pg 8, lines 250-253).*

Comment 17: The same for the ICBP Module 4 data coding rules.

Response: *We have expanded upon our description, and referred readers to the full paper should they wish to review it further: "Where there are missing or incomplete data, or multiple responses, we will apply the ICBP Module 4 data coding rules, which provide a hierarchy for information sources.[23] For full details, please refer to the original publication.[23]" (pg. 8, lines 253-255)*

Comment 18: "Interaction terms between rurality status and key predictors will be included." Because researchers are inclined to remove interaction terms that are not statistically significant, you don't get a good idea of different situations in different main groups. Please consider stratified analysis in rural and urban groups as an alternative.

Response: *We thank the reviewer for this suggestion. Rurality is a key variable of interest in this study, and interaction terms are included to formally assess whether the association between selected predictors and the main outcomes differs between rural and urban populations. We believe this approach is preferable to fitting separate stratified models because this approach allows the full dataset to be utilised and facilitates direct comparison of predictor-outcome associations across rurality groups. Where interactions are found to be important, we will present rural- and urban-specific estimates to aid interpretation.*

Comment 19: Poisson regression. It is not entirely clear to me why you use this instead of logistic regression.

Response: *We agree that logistic regression is most appropriate. This was an error and has now been corrected to read logistic regression (pg 8, line 276).*

Comment 20: Page 10 line 20: remove "of".

Response: *This has been done.*

Comment 21: Page 10 line 24: what are "delayed predictors"?

Response: *This has been revised in response to reviewer 1's comment: "The inclusion of different multiple cancer types, which allows for analysis to identify whether predictors of delays differ by both geographic region and cancer type." (page 10, lines 325-327)*
