# Supplementary material for: Identifying the predictors of time to diagnosis and treatment for people with cancer: a protocol for a cross-sectional survey supported by linked cancer registry data
Source: BMJ Open. 2026 Oct 28;16(10):e123201. doi: 10.1136/bmjopen-2026-123201 (PMC13723840; doi:10.1136/bmjopen-2026-123201)
Supplement: Supplementary file 2 [file bmjopen-16-10-s001.docx]

Manuscript Title: Identifying the predictors of time to diagnosis and treatment for people with cancer: A protocol for a cross-sectional survey supported by linked cancer registry data

**Supplementary File 1**

Patient Questionnaire.

**About your cancer diagnosis and treatment**

In this section, we will ask questions about your cancer diagnosis and treatment. If you have had cancer more than once, for all questions, please consider your **[insert specific tumour type]** cancer diagnosis.

**Which of the following best describes the events which led to your diagnosis of cancer?**

- I had symptoms/I noticed a bodily change and went to see a doctor (e.g. General Practitioner)
- I had symptoms/I noticed a bodily change and went/was taken to the emergency department
- I had seen a doctor with symptoms, but went/was taken to the emergency department when things worsened
- I was being investigated for another health problem during which time the cancer was discovered
- Other (please specify)

**When did you first notice any symptoms that led to being tested for cancer? Please write down your best estimate of the date. If you cannot remember the exact date, you can fill in the month and the year.**

- ___/___/____ (Use DD/MM/YYYY format)
- This is not applicable to me (e.g., I had no symptoms)

**What type of health professional did you first talk to about your symptoms?**

- General Practitioner
- Nurse
- Other (please specify)

**For approximately how long did you have health concern(s) or symptom(s) before contacting a health professional?** Please think of the first visit to a health professional, not revisits. If you were being investigated for another problem, please think about how long you had the symptoms or concerns that led to these investigations.

- Less than 1 week
- 1-2 weeks
- 2-3 weeks
- 3-4 weeks
- 1-2 months
- 3-5 months
- 6-12 months
- More than 12 months
- This is not applicable to me (e.g. I had no symptoms)

**Once you decided to have contact with a health professional about your health concern(s) or symptom(s), how long did it take to get an appointment?** Please think of the first visit to the health professional to discuss your concern(s) or symptom(s) and select one option below.

- There was no waiting time because I went/was taken to the emergency department
- Same day/next day
- Within 1 week
- 1-2 weeks
- 3-4 weeks
- More than 4 weeks
- This is not applicable to me (e.g. I had no symptoms)

**What was the date of your first appointment with a health professional (e.g. GP) about your health concern(s) or symptom(s).** If you cannot remember the exact date, you can fill in the month and the year

- ___/___/____ (Use DD/MM/YYYY format)
- This is not applicable to me (e.g. I had no symptoms)

**Was this appointment in person, by phone (i.e. telehealth) or by video?** *Please select one option below.*

- In person
- Phone
- Video
- Don’t remember / don’t know

**Who was the health professional you saw at this first appointment?** *Please select one option below.*

- Your usual GP
- A different GP at the clinic you usually go to
- A GP at a clinic you hadn’t attended before
- An emergency room doctor
- Other health professional (please specify)
- This is not applicable to me (e.g. I had no symptoms)
- Don’t remember / don’t know

**How many different GPs did you visit before your cancer was diagnosed?** *Please select one option below.*

- I did not see a GP
- 1
- 2
- 3 or more

**How many GP appointments did you have before you were diagnosed with cancer?** *Please select one option below.*

- I did not see a GP
- 1
- 2
- 3
- 4+

**Prior to your diagnosis, did you have a regular GP?** *Please select one option below.*

- I had a regular GP
- I saw different GPs at the clinic I usually go to
- I did not have a regular GP or regular clinic at the time of my diagnosis

**Prior to your diagnosis, did you participate in organised cancer screening programs?** Organised cancer screening programs include the National Bowel Cancer Screening Program, National Breast Cancer Screening Program, or the National Cervical Screening Program.

*Please select one option below.*

- Yes, I have participated consistently in programs that I have been invited to participate in
- Yes, I have participated in programs I have been invited to, but only sometimes
- No, I have not participated in programs I have been invited to
- Not applicable (I have never been invited to participate in a cancer screening program)

**When were you told of your cancer diagnosis?** *Please provide the day, month and year. If you cannot remember the day, please provide month and the year*

- ___/___/____ (Use DD/MM/YYYY format)
- Don’t remember/don’t know

**Which health professional informed you that you definitely had cancer?** *Please select all that apply.*

- GP
- Surgeon
- Oncologist
- Hospital doctor
- Not sure / don’t remember
- Other (please specify)

_____________________________________

**Do you think your cancer could have been diagnosed sooner?** *Please select one option below.*

- Yes
- No
- Possibly

**If yes or possibly, why do you think your cancer could have been diagnosed sooner?**

**How did you feel about the cancer diagnosis services offered in your area?** *Please select one option below.*

- I was / am satisfied with the cancer services offered in my area
- I was / am unsatisfied with the cancer services offered in my area
- Unsure / prefer not to say

**If unsatisfied, how could the services in your local area be improved?**

**How long did you have to wait from when you were referred, to when you were seen, by a doctor or hospital clinic for your first appointment?** *Please select one option below.*

- Less than two weeks
- More than two weeks but within four weeks
- More than a month
- Not sure / cannot remember
- Other (please specify)

**If more than two weeks, was this due to:**

- Personal decision to wait
- Specialist waiting times
- Hospital waiting times
- Other (please describe)

**What was the date of your first appointment with a doctor, involved in investigating and/or treating your cancer, to whom you were referred?** *Please select one option below.*

- ___/___/____ (Use DD/MM/YYYY format)
- This is not applicable to me (e.g. I had no symptoms)

**Was this appointment in person, by phone or by video?** *Please select one option below.*

- In person
- Phone
- Video
- Don’t remember / not know

**Which doctor/s were in charge of your care?** *Please select all that apply.*

- Surgeon
- Medical oncologist
- Radiation oncologist
- Gynaecologist
- Urologist
- GP
- I am not sure who is / was in charge of my care
- Other (please specify)
- Other medical doctor (please specify)

**We know it can be overwhelming when you are first told you have cancer. However, if you can recall, did the doctor explain your cancer diagnosis in a way you could understand?** *Please select one option below.*

- Yes, definitely
- Yes, to some extent
- Not really
- No, not at all
- Don’t remember / not sure

**During the process of receiving your cancer diagnosis, how available were health services, professionals, or supports when you needed them?** *Please select one option below.*

- Very available
- Somewhat available
- Somewhat unavailable
- Unavailable
- Don’t remember / not sure

**Did financial barriers affect your ability to receive all the information and supports you needed during the process of being diagnosed with cancer?** *Please select one option below.*

- They had no impact on my ability to receive all the information and supports I needed
- They had an impact on my ability to receive all the information and supports I needed
- Unsure / prefer not to say

**When decisions were made about your cancer care and treatment, were you involved as much as you wanted to be?** *Please select one option below.*

- Yes, definitely
- Yes, to some extent
- No
- I was not well enough
- I did not want to be involved in these decisions
- I don’t believe there were any relevant decisions that I should have been involved in
- Not sure / don’t remember

**Did you have any treatment for your cancer (e.g. Surgery, chemotherapy, radiation therapy, or hormonal therapy?)** *Please select one option below.*

- No
- Yes – You can skip the next question.

**If you did not have any treatment for your cancer, what were the main reasons for this?** *Please select all that apply***.**

- Treatment wasn’t suggested or recommended by my doctor
- I chose not to have treatment
- My treatment options have not been discussed yet
- My treatment hasn’t commenced yet
- My cancer is being monitored but not actively treated
- Another reason (please describe)

| **Have you had any of the following treatments aimed at removing or reducing cancer yet?** *If so, can you please estimate the date this treatment started? If you cannot remember the exact date, you can fill in the month and the year.* | | | |
| --- | --- | --- | --- |
|  |  | | **Date of treatment** *(Give first date if series of treatments)* |
|  | Yes | No |  |
| **Surgery**  *Please tick in the yes or no box.* |  |  | D \| D \| \| M \| M \| \| Y \| Y \| Y \| Y |
| If yes, what was the name of the facility where you had surgery?  Name______________________________ Suburb/town______________________ Postcode_________ | | | |
| **Chemotherapy**  *Please tick in the yes or no box.* | Yes | No | D \| D \| \| M \| M \| \| Y \| Y \| Y \| Y |
|  |  |  |  |
| If yes, what was the name of the facility where you had/have chemotherapy?  Name_________________________ Suburb/town______________ Postcode______ | | | |
| **Radiotherapy (also called radiation therapy)** *Please tick in the yes or no box.* | Yes | No | D \| D \| \| M \| M \| \| Y \| Y \| Y \| Y |
|  |  |  |  |
| If yes, what was the name of the facility where you had/have radiotherapy?  Name_________________________ Suburb/town______________ Postcode______ | | | |
| **Other treatment?** *Please tick in the yes or no box and describe the treatment.*  *Please specify:* | Yes | No | D \| D \| \| M \| M \| \| Y \| Y \| Y \| Y |
|  |  |  |  |
| If yes, what was the name of the facility where you had/have this treatment?  Name_________________________ Suburb/town______________ Postcode______ | | | |
| 🞏 **Treatment not started yet** | | | |

**Did any member of your healthcare team discuss with you the possibility of taking part in cancer research (clinical trials)?** *Please select one option below.*

- Yes, and I was comfortable with the discussion
- Yes, but I was not comfortable with the discussion
- No, but I would have liked them to have discussed this
- No, and I am glad they did not discuss this
- Don’t remember / not sure

**During or after your treatment, did you see an allied health professional?** *E.g., dietitian, physiotherapist. Please select one option below.*

- Yes
- No
- Don’t remember / not sure

**If yes, of the following did you see?** *Please select all that apply.*

- Physiotherapist
- Occupational therapist
- Dietitian
- Psychologist
- Social worker
- Exercise physiologist
- Speech pathologist
- Other (please specify)

**Did financial barriers affect your ability to start cancer treatment?** *Please select one option below.*

- They had no impact on my ability to start cancer treatment
- They impacted my ability to start my treatment
- Unsure / prefer not to say

**For the duration of your treatment, where did you stay most of the time?** *Please select one option below.*

- At home
- With family and / or friends
- Organised accommodation
- Other (please specify)

**During your treatment, was there a time where you had to stay away from home?** *Please select one option below.*

- No, I stayed at home
- Yes, with family and / or friends
- Yes, in organised accommodation
- Yes, other (please specify)

**What mode of transport did you take to appointments and / or treatment?**

- Public transport (e.g., bus or train)
- Drove myself to appointments / treatment
- Friend drove me to appointments or treatment
- Family drove me to appointments or treatment
- Taxi / rideshare
- Other (please specify)

**Were there other barriers than prevented you commencing your treatment (e.g. caring responsibilities for minor children or employment barriers)?**

- No
- Yes, please specify

**Did staff consider your personal circumstances when arranging your appointment times?** For example, work, caring for family members, travel, or other commitments you have.

- Yes, definitely
- Yes, to some extent
- No
- Don’t’ remember / not sure

**When you had finished your most recent treatment to remove or reduce the cancer, were you given a written plan that included information about your follow-up care over the next year?** By follow-up care, we mean more than simply a list of medical appointments.

- Yes, I was given this information
- Yes, but I would have liked more
- No
- I have not finished treatment yet
- Don’t remember / not sure

**What type of healthcare professional is mainly in charge of your follow-up care?** If there is more than one doctor, please select all that apply.

- Surgeon
- Haematologist
- Radiation oncologist
- GP
- Medical oncologist
- Nurse
- I am not sure who is in charge
- Other (please specify)

**Which hospital or clinic has the most involvement in your follow-up care?** This could be the location of the doctor in charge of your follow-up care, or the hospital / clinic where you have most of your follow-up appointments.

Please write name of hospital / clinic and the town or suburb of where it is located.

Name of hospital or clinic

Name of town or suburb

- Don’t know / not sure

**Were you given the contact details for someone you could call if you had any complications or care needs?**

- Yes
- No
- Don’t remember / not sure

**About You**

In this section we will ask about yourself. If you have had cancer more than once, for all questions, please consider your **[insert specific tumour type]** cancer diagnosis.

**Looking back on the 2 years before you were diagnosed with cancer, would you say your general health was:**

- Very good
- Good
- Fair
- Poor
- Very poor

**Are you of Aboriginal or Torres Strait Islander origin?**

- No
- Yes, Aboriginal
- Yes, Torres Strait Islander
- Yes, both Aboriginal and Torres Strait Islander

**What is your highest level of education?**

- Primary school
- Some secondary school
- Completed secondary school
- Certificate or diploma
- Bachelor’s degree
- Postgraduate degree (e.g., Masters, PhD)
- I did not have the opportunity to complete any type of schooling

**What gender do you identify as?**

- Male
- Female
- Self-described (please specify):
- Prefer not to say

**How would you describe your sexual orientation?**

- Straight or heterosexual
- Gay, lesbian or homosexual
- Bisexual or pansexual
- Asexual
- Another sexual orientation (please describe)
- Don’t know
- Prefer not to say

**What is your current relationship status?** Please select the option that most closely reflects your relationship status.

- Single or never married
- In a relationship but not living with a partner
- De facto or living with a partner
- Married
- Separated or divorced
- Widowed

**Were you pregnant or postpartum (delivered a baby within the preceding 12months from your diagnosis) at the time of your cancer diagnosis?**

- Yes
- No
- Does not apply to me
- Prefer not to say

**Are you responsible for raising any children aged under 18?**

- Yes
- No
- Prefer not to say

**Before you received a diagnosis of cancer, what was your employment status?** Select all that apply.

- Working full-time
- Working part-time
- Working casually
- Looking for work / unemployed
- Student (full-time or part-time)
- Not working and not looking for work
- Retired
- Other (please specify)

**In which country were you born?**

- Australia
- England
- New Zealand
- India
- Philippines
- Vietnam
- Italy
- South Africa
- Malaysia
- Scotland
- Prefer not to say
- Other (please specify)

**What cultural background or ethnicity do you identify with?**

- None
- Australian
- English
- Irish
- Scottish
- Chinese
- Italian
- Indian
- German
- Greek
- Vietnamese
- Other (please specify)
- Prefer not to say

**What languages are spoken frequently at home or between you and your family?**

**What countries were your parents born in?** tick all that apply

- Australia
- England
- New Zealand
- India
- Philippines
- Vietnam
- Italy
- South Africa
- Malaysia
- Scotland
- Prefer not to say
- Other (please specify)

**Did you have private health insurance at the time of your cancer diagnosis?**

- No
- Yes, hospital cover only
- Yes, hospital and extras
- Don’t remember / not sure

**Do you have any of the following existing health problems other than cancer?**

- Heart disease
- Arthritis
- Diabetes
- Asthma
- Depression
- Anxiety
- Chronic pain
- Other, please specify

**How would you describe your current smoking behaviour?**

- I have never smoked
- I used to smoke
  - I smoked for about ____ years or _____ months
  - When I smoked, I had about ____ cigarettes per week
- I currently smoke
  - I smoked about ____ cigarettes per week
